# Neuroinflammatory Findings of Corneal Confocal Microscopy in Long COVID-19 Patients, 2 Years after Acute SARS-CoV-2 Infection

**DOI:** 10.3390/diagnostics13203188

**Published:** 2023-10-12

**Authors:** Pilar Cañadas, Leonela Gonzalez-Vides, Marta Alberquilla García-Velasco, Pedro Arriola, Noemí Guemes-Villahoz, Jose Luis Hernández-Verdejo

**Affiliations:** 1 Optometry and Vision Department, School of Optometry, Complutense University of Madrid, 28037 Madrid, Spain; leogon01@ucm.es (L.G.-V.); martaagv22@gmail.com (M.A.G.-V.);; 2 Education Faculty, University of Costa Rica, San José 11501-2060, Costa Rica; 3 Department of Ophthalmology, Hospital Clínico San Carlos, 28040 Madrid, Spain

**Keywords:** corneal confocal microscopy, corneal nerve plexus, COVID-19, Long-COVID, dendritic cells

## Abstract

Objective: To describe corneal confocal microscopy findings in patients with long COVID-19 with persistent symptoms over 20 months after SARS-CoV-2 infection. Design: A descriptive cross-sectional study that included a total of 88 patients; 60 patients with Long COVID-19 and 28 controls. Long COVID-19 diagnosis was established according to the World Health Organization criteria. Corneal confocal microscopy using a Heidelberg Retina Tomograph II (Heidelberg Engineering, Heidelberg, Germany) was performed to evaluate sub-basal nerve plexus morphology (corneal nerve fiber density, nerve fiber length, nerve branch density, nerve fiber total branch density, nerve fiber area, and nerve fiber width). Dendritic cell density and area, along with microneuromas and other morphological changes of the nerve fibers were recorded. Results: Long COVID-19 patients presented with reduced corneal nerve density and branch density as well as shorter corneal nerves compared to the control group. Additionally, Long COVID-19 patients showed an increased density of dendritic cells also with a greater area than that found in the control group of patients without systemic diseases. Microneuromas were detected in 15% of Long COVID-19 patients. Conclusions: Long COVID-19 patients exhibited altered corneal nerve parameters and increased DC density over 20 months after acute SARS-CoV-2 infection. These findings are consistent with a neuroinflammatory condition hypothesized to be present in patients with Long COVID-19, highlighting the potential role of corneal confocal microscopy as a promising noninvasive technique for the study of patients with Long COVID-19.

## 1. Introduction

Coronavirus-2019 (COVID-19) disease caused by severe acute respiratory syndrome coronavirus (SARS-CoV-2) has shown clinical manifestations in virtually all organs, including a broad range of neurological symptoms, such as headache, fatigue, loss of taste and smell, brain fog, and neuropathic pain [1].

A variable proportion of patients who have overcome SARS-CoV-2 infection may experience long-term effects of the infection, known as Long COVID-19. Long COVID-19 (LC-19) is also known by other names such as post-COVID conditions (PCC), post-acute sequelae of SARS-CoV-2 infection (PASC), long-haul COVID, and chronic COVID. LC-19 symptoms are complex and very heterogeneous, which may explain the lack of consensus on its definition to date. According to the Delphi consensus by the World Health Organization, LC-19 occurs in individuals with a history of probable or confirmed SARS-CoV-2 infection, usually within 3 months of onset, with symptoms lasting for at least 2 months which cannot be explained by an alternative diagnosis. The most commonly presenting symptoms include fatigue, respiratory distress, and cognitive dysfunction. Symptoms may also fluctuate or relapse over time. However, the consensus definition is likely to evolve as knowledge increases [2]. Recent studies have shown a negative impact on cognitive function, especially significant linguistic–cognitive and visual attention impairment [3,4]. Consequently, people with the LC-19 condition suffer from a decreased quality of life [5] and physical activity levels, mostly due to intense daily fatigue that can occur even during daily activities [4].

The underlying mechanism by which SARS-CoV-2 affects the nervous system is still unknown. However, it appears that an innate immune response and adaptive immunity are involved [6]. Recently published studies associate this condition with small fiber neuropathy (SFN) and peripheral neuropathy. Peripheral neuropathy and autonomic involvement are characterized by a selective alteration of small, thinly myelinated nerve fibers such as A-fibers, and unmyelinated C-fibers. Corneal nerve fiber loss, indicating neurodegeneration, may also be associated with other diseases such as fibromyalgia, diabetic neuropathy, and even Alzheimer’s disease [7,8,9].

The cornea, as one of the most innervated tissues in the human body, [10] receives heterogeneous sensory nerves from the ophthalmic branch of the trigeminal nerve. In addition to these sensory fibers, the cornea also receives a small supply of autonomic sympathetic nerve fibers, which originate from the cell bodies of the superior cervical ganglion [11,12,13]. Thus, the cornea is an interesting tissue to noninvasively image the nerves of. In vivo confocal microscopy (IVCM) is a useful tool to assess the integrity of the peripheral nervous system, even in neurodegenerative diseases [14]. In addition, the cornea, in particular its basal epithelium, is populated by resident immune cells, known as dendritic cells (DCs) [15]. The function of DCs is to be immune sentinels, which bridge the innate and adaptive immune responses, [16] and also contribute to maintaining corneal nerve homeostasis [17].

Microneuromas have been identified in the sub-basal nerve plexus and stromal nerves in patients after SARS-CoV-2 infection [18]. In fact, microneuromas may represent the result of nerve damage and subsequent nerve regeneration [19].

In the global effort to control the spread of SARS-CoV-2, the development of COVID-19 vaccines have been important. These vaccines have demonstrated remarkable efficacy in preventing severe disease, hospitalization, and death. However, concerns have emerged regarding potential adverse effects, including neurological complications [20]. Understanding the impact of COVID-19 vaccines on neuroinflammation and their influence on patients with LC-19 is one of the most important issues to guide clinical management and improve patient outcomes.

There are only a few reports to date that have evaluated corneal innervation in COVID-19 patients [21]. Some have demonstrated reduced corneal nerve fibers and increased DCs in patients with active COVID-19 and in patients with LC-19 3–4 months after infection [22]. However, none of these studies have evaluated patients with persistent symptomatology nearly 2 years after acute infection.

Therefore, the purpose of this study is to depict corneal confocal microscopy findings in LC-19 patients with persistent symptoms over 20 months after infection.

## 2. Material and Methods

A descriptive cross-sectional study was conducted at Hospital Clinico San Carlos (HCSC) in Madrid, Spain. The study was approved by the HCSC’s Clinical Research Ethics Committee and was conducted in accordance with the Declaration of Helsinki. Written informed consent was obtained from all subjects.

The inclusion criteria were as follows: subjects over 18 years of age with LC-19. Those subjects with concomitant eye diseases, including ocular surface disease, glaucoma, or any other ocular disease that required topical ocular treatments were excluded. History of systemic disease before infection, and contact lens wearers were also excluded. Subjects undergoing ocular surgery in the previous 6 months or those lacking consent to participate in the study were also excluded. Subject demographic data, medical history, history of SARS-CoV-2 infection, and vaccination regimen against COVID-19 were collected.

A group of volunteers pre-pandemic over 18 years of age, non-contact lens wearers with no systemic disease, no eye diseases including ocular surface disease, glaucoma, or any other ocular disease that required topical ocular treatments served as the control group.

All of the LC-19 patients enrolled were diagnosed based on World Health Organization indications and following the clinical guidelines [23].

A questionnaire in accord with the Long COVID-19 guideline developed by the National Institute for Health and Care Excellence (NICE), the Long-COVID Questionnaire, was administered to identify persisting symptoms at 4 weeks, 12 weeks, and actually after the diagnosis of COVID-19 [24].

### 2.1. In Vivo Confocal Microscopy

In vivo confocal microscopy images were obtained using the Heidelberg Retina Tomograph II in combination with the Rostock Cornea Module (RCM) (Heidelberg Engineering, Heidelberg, Germany). Prior to microscopic imaging, a drop of Viscotears gel (Novartis, North Ryde, Australia) was applied into a Tomo Cap (Heidelberg Engineering, Heidelberg, Germany), which was then positioned on the lens of the microscope. Then, another drop of gel was placed on the tip of the Tomo Cap.

The examination was performed under topical anesthesia. (Colircusí Anestésico Doble^®^; Alcon Laboratories Inc., Barcelona, Spain). The RCM was set in “volume mode” to record a video of the different corneal layers down to a depth of 80 μm. Then, at least three nonoverlapping scans were obtained, from the epithelium to the anterior stroma at 80 μm depth using volume scans. The video with the least artifacts was selected for evaluation. The selected video was automatically converted into a series of two-dimensional images. All images from each scan were reviewed and the three best images (images that were out of focus, not in a consistent plane, or showed artifactual compression lines were excluded from the analysis) containing the sub-basal nerve plexus were manually selected and analyzed in each eye, and the mean was expressed. The participant was looking at a near-fixation point to ensure that the image was captured at the center of the cornea. This fixation point was always the same. Each image had a resolution of 384 × 384 pixels, showing a coronal section of the sub-basal nerve plexus of 400 × 400 μm.

### 2.2. Image Analysis

A single experienced, masked examiner analyzed all confocal images obtained in both groups. All IVCM images were coded to avoid bias.

#### 2.2.1. Nerve Plexus Morphology

Three high-quality images of the sub-basal nerve plexus in each eye were selected and analyzed using ACCMetrics software version 3 (University of Manchester, Manchester, UK).

The morphology parameters of the sub-basal nerve plexus were as follows: corneal nerve fiber density (CNFD) (number of nerve fibers per square millimeter), nerve fiber length (CNFL) (total length of nerves in millimeters per square millimeter), nerve branch density (CNBD) (number of primary branch points on the main nerve fibers per square millimeter), and nerve fiber total branch density (CTBD) (total number of branch points per square millimeter).

Other morphological changes of nerve fibers, including microneuromas, were recorded.

#### 2.2.2. Dendritic Cell Density and Area

**Figure 1:**
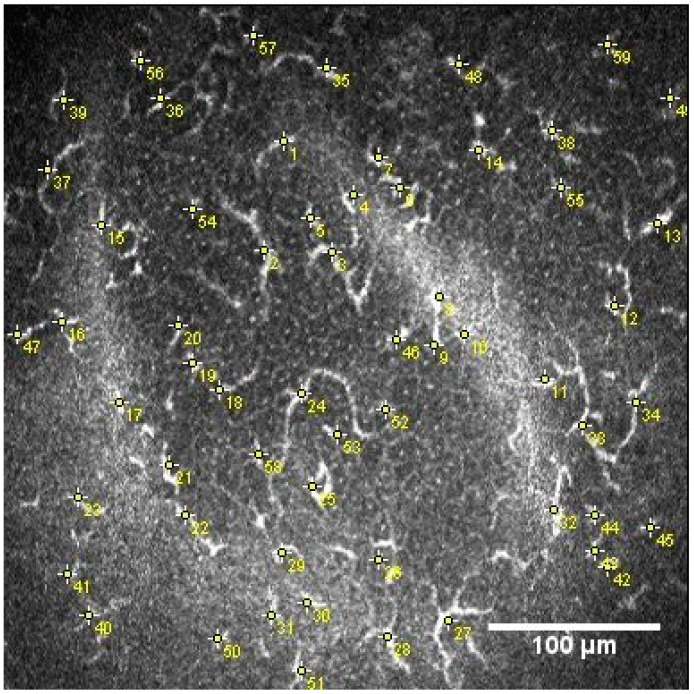
Dendritic cell count.

The DCs, identifiable in the sub-basal nerve plexus from their distinctive characteristics (bright cell bodies with dendritic structures), were manually numbered using the multipoint tool of the Image J software and then the DC density (cells/mm^2^) was calculated, as shown in Figure 1. In the DC quantification process, the image contrast was modified to make the hyperreflective bodies of these cells more visible [25].

**Figure 2:**
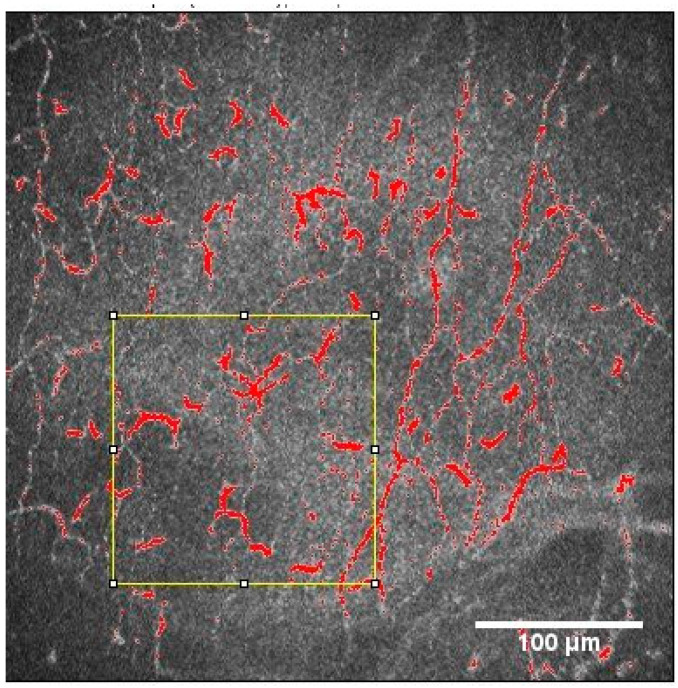
Dendritic cell area analysis.

To measure the DC area (DCA), DC perimeter (DCP), and circularity coefficient (CC) on IVCM images, the threshold function of ImageJ software (http://imagej.nih.gov/ij/, accessed on 25 February 2023) was used. First, the 10 representative DCs were selected based on the original IVCM image. Then, the threshold function was applied to highlight the DCs and the area. [9] This is shown in Figure 2.

### 2.3. Statistical Analysis

Statistical analysis was performed using SPSS version 21.0 (Chicago, IL, USA) software. The normality of the data distribution was tested via the Kolmogorov–Smirnov test. The Wilcoxon test was employed to assess the statistical significance of the differences between paired samples. A *p* ≤ 0.05 was considered statistically significant. Data are expressed as mean and standard deviation (SD).

The relationships between corneal confocal microscopy numeric variables were analyzed using the Spearman correlation coefficient, and *p* ≤ 0.001 was considered statistically significant.

## 3. Results

### 3.1. Confocal Microscopy Findings

In total, 88 participants were included, with 60 in the LC-19 group and 28 in the control pre-pandemic group.

The comparisons between groups are shown in Table 1. We found significantly lower values in of CNFD, CNFL, and CTBD in LC-19 patients and significantly higher values of DCA, DCP and CC.

**Figure 3:**
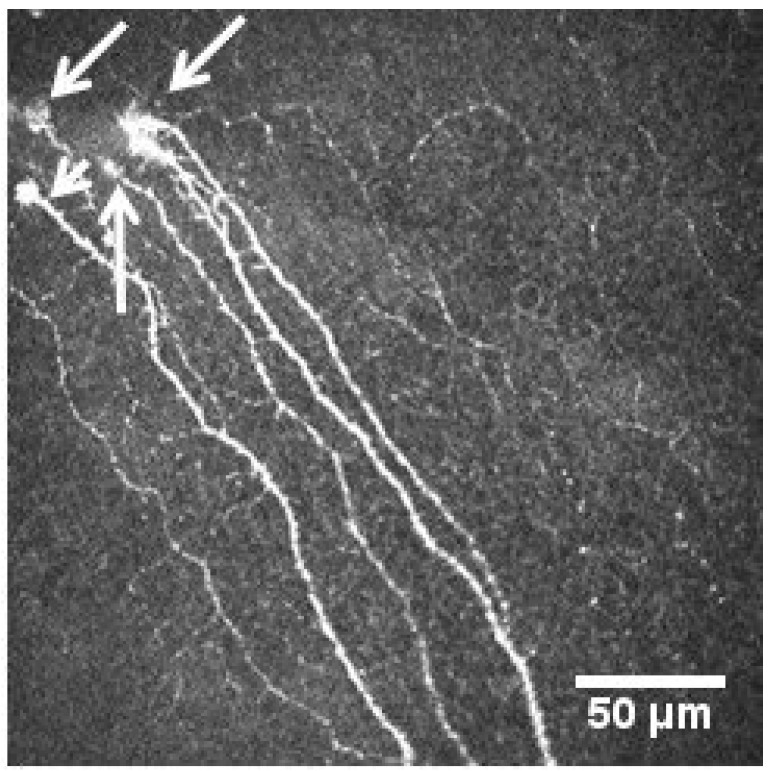
Corneal sub-basal nerve plexus with visible microneuromas marked by with arrows.

We also found the presence of microneuromas in nine eyes (15%) in the LC-19 group (Figure 3).

**Figure 4:**
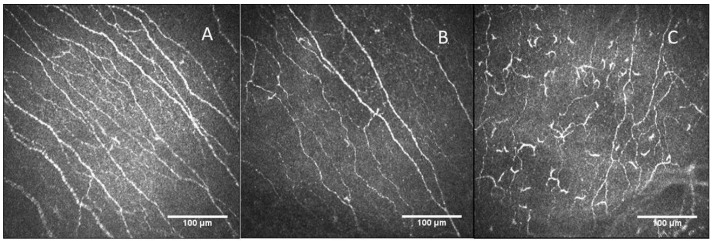
Representative CCM images: (**A**) Corneal nerve plexus in control patient. (**B**) Corneal nerve plexus in Long COVID-19 patients. (**C**) Dendritic cell representative image in Long COVID-19 patient.

Sub-basal nerve plexus images of the central cornea in the control and LC-19 groups, and DCs in the LC-19 group showed some distinctive features (Figure 4). 

### 3.2. Relationship between Corneal Confocal Microscopy Morphology Parameters in LC-19 Patient

Positive significant correlations were found between CNBD, CTBD, and CNFD (Rho = 0.62, *p* < 0.001; Rho = 0.45, *p* < 0.001), and between CNFD and CNFL (Rho = 0.59, *p* < 0.001). Other positive significant correlations were found between the following: DCD and their morphological parameters DCA (Rho = 0.88; *p* < 0.001), DCP (Rho = 0.88; *p* < 0.001) and CC (Rho = 0.58; *p* < 0.001).

Although not significant, a positive correlation was found between months after infection and CNFD (Rho = 0.11; *p* = 0.40), and a negative correlation was found between DCD and months after the infection (Rho = −0.07; *p* = 0.59).

### 3.3. Corneal Confocal Microscopy Parameters in Vaccinated and Unvaccinated LC-19 Patients

Table 2 shows the descriptive data of both groups. Lower CNFD, and higher DCD, DCA, and DCP in the unvaccinated group were found, but there were no significant differences between groups.

## 4. Discussion

Long COVID has shown an impact on the nervous system. In particular, our results have revealed differences in corneal innervation determined using corneal confocal microscopy. We observed lower corneal nerve densities, shorter corneal nerves, and lower branch densities in patients with LC-19. Additionally, our study also showed higher DC activation, the areas of which were greater. Interestingly, microneuromas were also found in several Long COVID-19 patients. Regarding the vaccination influence, our results also showed lower nerve density, higher DC density, and more active DCs in unvaccinated patients, but these comparisons were non-significant.

A reduction in corneal nerve parameters has been reported in previous studies performed in patients affected with COVID-19. Mirza et al. [26] also found a reduction in corneal nerve density, corneal nerve length, and total branch density in post-COVID-19 patients one month after infection, in patients both with and without neurological symptoms. Although none of the included patients had any visual symptoms, it appears that a history of COVID-19 might have influenced the subclinical changes found in the corneal nerve plexus. Bitirgen et al. [22] also found a decrease in nerve plexus parameters in patients with Long COVID-19 with a persistence of symptoms 3–4 months after acute infection. However, our work found a long-term persistence of these features (a decrease in corneal nerve density, shorter-length nerves, and a lower branch density) 20 months after acute infection.

The underlying mechanism for corneal nerve involvement is unclear. Inflammatory mediators and biochemical cascades may be implicated in this condition. Neuropathological studies have shown SARS-CoV-2 in the cerebrum, cerebellum, cranial nerves, olfactory bulb, and olfactory epithelium, with associated microglial activation and lymphoid inflammation [27]. Furthermore, COVID-19 infection has been associated with post-infectious immune-mediated peripheral neuropathy [28], whereas an improvement in neuropathy has been observed following plasma exchange [29].

We also found that nine LC-19 patients (15%) presented with corneal microneuromas, whereas this finding was not present in either of the images of any of the patients in the control group. Microneuroma formation is not fully understood. However, it has been described post-injury. Sprouts will grow from the proximal nerve stump advancing distally, and those sprouts may grow extraneurally, forming microneuromas [19]. In addition, a neuroinflammatory process has been related to microneuroma presence [30], which is the mechanism also present in LC-19 patients. Microneuromas in the human cornea have been also described in several other pathological conditions such as postkeratoplasty, neuropathic pain, dry eye, etc. [31,32,33]. However, none of the patients with LC-19 had a previous history of any of these conditions. Therefore, microneuromas may represent the result of nerve damage and subsequent nerve regeneration. These recovery signs could also be shown via positive correlations seen in LC-19 patients with months after infection and CNFD, and the negative correlation between months after infection and DCD. Although these correlations were not significant, further studies should be performed with a long follow-up to corroborate these findings.

Our study showed an increase in activated DCs in patients with LC-19 and a decrease in nerve density, nerve branch density, and nerve length, these findings being consistent with an inflammatory and innate immune response. Immature DCs are located in the corneal periphery. In a number of immune-mediated and inflammatory conditions, after antigen take-up and processing, DCs become mature, with migration to the central cornea [34,35,36]. Our results showed an increase in DC areas and their morphological parameters in LC-19 patients. Bitirgen et al. [22] found an increase in DC density in patients with LC-19 and neurological symptoms 3–4 months after the acute infection, but did not measure the DC area. However, they found that the DCs were active due to the cell’s morphology. Our results confirm the activation of DCs in an objective way, due to the larger area, as is shown in Figure 4. As is known, the role of DCs is to be immune sentinels, and also contribute to maintaining corneal nerve homeostasis [16,17]. Thus, these immune and inflammatory pathways may contribute to the nerve degeneration seen in LC-19 patients, 20–24 months after acute infection. Further studies are needed to support this theory.

To the best of our knowledge, there was no specific research or established direct relationship between Long COVID-19 in unvaccinated patients and an increase in dendritic cells in the cornea. Our results showed lower corneal nerve density, higher dendritic cell density and that those cells were more active; however, these results were not significant. Some research suggests that COVID-19 vaccination may lead to an increase in dendritic cells in the cornea, potentially enhancing local immune responses and protection against viral infections [20]. However, any connection between Long COVID-19 and unvaccinated individuals in our study should be viewed with caution, as the sample size is very small.

The use of IVCM to identify damage in SFN and in degenerative disorders of the central and peripheral nerve systems is well described and accepted. IVCM is a noninvasive imaging technique that allows the direct visualization of the corneal structure, including the corneal sub-basal nerve plexus in vivo. For this reason, IVCM has been used to identify small nerve fiber damage in several peripheral neuropathies, such as diabetic neuropathy, and in immune-mediated SFN such as amyloidosis [8,37,38,39].

Our study has several limitations; it is a cross-sectional study so we need a long follow-up to evaluate the possible changes in corneal confocal findings. The findings after treatment could be interesting, so these could be the future lines of investigation.

## 5. Conclusions

In conclusion, our results suggest that there is an alteration of corneal nerve parameters and an increase in DC activation in LC-19 patients 20–24 months after infection. These findings are consistent with a neuroinflammatory condition that has been hypothesized to be present in Long-COVID-19 patients. Our results must be confirmed with a follow-up to observe the possible changes in LC-19 and the treatment influence on these patients.

## Figures and Tables

**Table 1:** Comparison of corneal nerve parameters and dendritic cell morphology in LC-19 patients and control group.

	Mean Age ± SD (Years)	Gender (F/M)	M.A.I ± SD (Months)	CNFD n/mm^2^	CNFL mm/mm^2^	CNBD n/mm^2^	CTBD n/mm^2^	DCD cells/mm^2^	DCA µm^2^	DCP µm	CC	Neuromas (Number of Patients)
Controls n = 28	39.4 ± 8.0	21/7	X	25.0 ± 10.7	33.9 ± 21.4	15.9 ± 4.2	56.8 ± 29.0	6.8 ± 9.9	30.5 ± 36.3	23.6 ± 28.8	0.1 ± 0.2	0
Long COVID-19 n = 60	46.5 ± 8.0	53/7	23.4 ± 7.7	16.3 ± 7.7	24.1 ± 17.3	13.0 ± 5.8	37.1 ± 20.9	13.2 ± 17.6	95.2 ± 101.6	77.5 ± 85.7	0.4 ± 0.5	9
*p* Value < 0.05 (*)	0.065	*		*	*	0.1091	*	0.2324	*	*	*	

CNFD: corneal nerve fiber density; CNFL: corneal nerve fiber length; CNBD: corneal nerve branch density; CTBD: nerve fiber total branch density; DCD: dendritic cell density; DCA: dendritic cell area; DCP: dendritic cell perimeter; CC: circularity coefficient; and M.A.I = months after infection. * Denote statistically significant.

**Table 2:** Descriptive data of vaccinated and unvaccinated LC-19 patients.

	YES	NO	*p* Values
Vaccinated N(%)	47.00 (78.33%)	13.00 (21.77%)	
Age (Years)	47.5 ± 7.8	42.7 ± 8.2	0.11
CNFD n/mm^2^	17.1 ± 7.8	13.6 ± 7.0	0.2192
CNBD n/mm^2^	24.8 ± 18.0	21.6 ± 14.6	0.7738
CNFL mm/mm^2^	12.6 ± 4.3	14.5 ± 9.7	0.7947
CTBD n/mm^2^	38.5 ± 22.0	32.3 ± 16.1	0.34
DCD n/mm^2^	10.1 ± 13.7	24.5 ± 24.9	0.1500
DCA µm^2^	88.9 ± 100.5	117.8 ± 106.2	0.3171
CDP µm	65.2 ± 67.7	121.8 ± 125.8	0.2869
CC	0.4 ± 0.6	0.4 ± 0.5	0.83
M.A.I (months)	23.7 ± 8.4	22.5 ± 4.6	0.56

CNFD: corneal nerve fiber density; CNFL: corneal nerve fiber length; CNBD: corneal nerve branch density; CTBD: nerve fiber total branch density; DCD: dendritic cell density; DCA: dendritic cell area; DCP: dendritic cell perimeter; CC: circularity coefficient; and M.A.I = months after infection. All data are expressed as mean ± SD.

## Data Availability

The data used to support the findings of this study are included within the article. Raw data are available from the corresponding, upon reasonable request.
